# A Semi-Supervised Transfer Learning with Grid Segmentation for Outdoor Localization over LoRaWans †

**DOI:** 10.3390/s21082640

**Published:** 2021-04-09

**Authors:** Yuh-Shyan Chen, Chih-Shun Hsu, Chan-Yin Huang

**Affiliations:** 1 Department of Computer Science and Information Engineering, National Taipei University, No. 151, University Rd., San Shia District, New Taipei City 23741, Taiwan; houngyin820912@gmail.com; 2 Department of Information Management, Shih Hsin University, No. 1, Ln. 17, Sec. 1, Muzha Rd., Wenshan Dist., Taipei City 116, Taiwan; cshsu@mail.shu.edu.tw

**Keywords:** outdoor localization, semi-supervised learning, deep learning, internet of thing (IoT), LoRaWAN

## Abstract

During the training phase of the supervised learning, it is not feasible to collect all the datasets of labelled data in an outdoor environment for the localization problem. The semi-supervised transfer learning is consequently used to pre-train a small number of labelled data from the source domain to generate a kernel knowledge for the target domain. The kernel knowledge is transferred to a target domain to transfer some unlabelled data into the virtual labelled data. In this paper, we have proposed a new outdoor localization scheme using a semi-supervised transfer learning for LoRaWANs. In the proposed localization algorithm, a grid segmentation concept is proposed so as to generate a number of virtual labelled data through learning by constructing the relationship of labelled and unlabelled data. The labelled-unlabelled data relationship is repeatedly fine-tuned by correctly adding some more virtual labelled data. Basically, the more the virtual labelled data are added, the higher the location accuracy will be obtained. In the real implementation, three types of signal features, RSSI, SNR, and timestamps, are used for training to improve the location accuracy. The experimental results illustrate that the proposed scheme can improve the location accuracy and reduce the localization error as opposed to the existing outdoor localization schemes.

## 1. Introduction

The location-based service (LBS) technology is very useful in many IoT-based location-aware applications [1,2,3,4,5,6]. The LBS has already been widely provided, such as navigation, location-based communication, and location-based data collection.

The LoRaWANs technology [3] has the advantages of the long-distance, low-cost, and low-power characteristics of LPWA (Low Power Wide Area) networks. A LoRaWAN-based GPS-free localization technique is an innovative way to provide the location information for the low-cost location-aware applications in the rural and urban outdoor environment. LoRa [3,4] is one of the LPWA communication technologies which uses the chirp spread spectrum modulation (CSS) to support long distance communication with low power consumption. These characteristics also provide an alternate way to support localization in the outdoor environment. When the LoRa packet from an end-node device is picked up by three or more gateways, the received signal strength indicator (RSSI) and the time different of arrival (TDOA) collected in LoRa gateways [5] can be used for localization.

Because the LoRa signals often go below the noise floor after penetrating barriers, the received signal is more sensitive to noises, interferences, and obstacles and hence the RSSI resolution or sensitivity of the path-loss might not be sufficient for outdoor localization. Therefore, RSSI, SNR, and timestamps are all considered as the input data for training so as to improve the accuracy of localization in the proposed scheme.

It is shown that the accuracy of localization in LoRa can be improved by machine learning technologies [7]. The machine learning technologies can be classified into supervised, semi-supervised, and unsupervised learning. Supervised learning is a machine learning task of learning a function from labelled training data consisting of a set of training examples. Semi-supervised learning is a class of supervised learning tasks from a small amount of labelled data with a large amount of unlabelled data [8,9,10]. Unsupervised machine learning is the machine learning task of inferring a function for unlabelled data. Since it is not feasible to collect all the datasets of labelled data in an outdoor environment, the semi-supervised transfer learning is adopted in the proposed scheme.

The deep learning is the application of artificial neural networks to learn tasks which contain more hidden layers. In deep learning, each level learns to transform its input data into a slightly more abstract and composite representation. The deep learning architecture is constructed with a greedy layer-by-layer method. The depth in deep learning is the number of layers through which the data are transformed. The learning architecture with many hidden layers is called as the deep neural network (DNN). The DNN [11] simulates the hierarchical structure of the human brain, processing data from low level to high level and gradually producing more and more semantic concepts. With multiple layers of nonlinear processing stages, DNN can extract complex structure and build an internal representation of big data. It is expected that the localization accuracy can be further improved by using deep learning models [1,12] and hence the deep learning architecture is adopted in the proposed scheme.

To improve existing localization works, we propose a novel outdoor localization scheme using a semi-supervised transfer learning for LoRaWANs. The semi-supervised DNN is used to derive the regression of the estimated location. The semi-supervised transfer learning is adopted because it is not feasible to collect all the labelled samples in an outdoor environment and the accuracy of the semi-supervised transfer learning is usually better than that of the unsupervised learning. The LoRaWAN technology is adopted in this paper because the LoRaWAN technology is one of the communication technologies that is usually used in the outdoor environment for IoT devices. With the low cost and long range characteristic of the LoRaWAN technology, a large area can be covered by very few gateways and hence the hardware cost can be greatly reduced.

In this paper, a novel grid segmentation scheme is proposed so as to generate a number of virtual labelled samples by figuring out the relationship between labelled and unlabelled samples. With the labelled-unlabelled samples relationship, we may repeatedly fine-tune our target model by adding more new virtual labelled samples so as to derive more accurate regression and achieve high localization accuracy. In short, the goal of this paper is to reduce the hardware cost of the outdoor localization for IoT devices and improve the localization accuracy of the existing works based on LoRaWANs. The experimental results illustrate that the proposed scheme effectively improves the average accuracy up to 91% in a large experimental area, and may reduce the average localization error up to 4 m in a small experimental area, compared with the existing outdoor localization results based on LoRaWANs.

The rest of this paper is organized as follows: Section 2 describes the related works. Section 3 describes the system model and defines the problem formulation. Section 4 proposed a semi-supervised transfer learning algorithm using grid segmentation. The experiment results are presented in Section 5 and the conclusions are finally given in Section 6.

## 2. Related Works

Some localization results are shown in Section 2.1 and the research motivation is discussed in Section 2.2.

### 2.1. Localization Results

This section introduces three classes of existing localization results [2,3,4,5,6,7,8,9,10,11,12,13,14,15,16,17,18,19,20,21,22,23]. The first class is the localization results only using RSSI, SNR, or timestamps for long-range IoT networks or LoRaWANs [2,3,4,5,6,13,14]. The second class is the indoor and outdoor localization using the supervised deep learning techniques [11,12,15,16,18,19]. The third class is the localization prediction results using the semi-unsupervised transfer learning techniques [8,9,10,20,21,22,23].

Firstly, some localization results are reported for long-range IoT networks or LoRaWANs [2,3,4,5,6,13,14] by only using one or more signal parameters, such as received signal strength indicator (RSSI), time different of arrival (TDOA), etc. Chiumento et al. [2] proposed a localization scheme in long-range ultra narrow band IoT networks, LoRa, or sigfox models, by using RSSI. In this work, RSSI has been used for fingerprinting localization, where RSSI measurement of GPS anchor nodes have been used. Lam et al. [3,4] proposed LoRa-based localization algorithms for the noisy outdoor environment by considering the RSSI to eliminate the Gaussian and non-Gaussian noise to select the non-noisy nodes. Based on LoRa localization report released by Semtech [5], they declared that they have an extensive experience for building a “time-doamin” based localization systems by considering the direct path energy, multipath correlation, and TDOA. Fargas et al. [6] proposed a GPS-free geolocation using LoRa technology by considering both RSSI and TDOA. Lam et al. [13] propose RSSI-based localization algorithms to reduce the effect of Gaussian and non-Gaussian noise in LoRa networks. Podevijn et al. [14] evaluate the localization accuracy, update probability, and update frequency for different trajectories (walking, cycling, and driving) and LoRa spreading factors. The median accuracy of the raw TDoA output data is 200 m. If the road map and movement speed are taken into account, the median accuracy is significantly improved to 75 m.

It is not easy to provide accurate localization results by using LoRa because of the following reasons. First, since the LoRa signals often go below the noise threshold after penetrating barriers, the localization based on received signal strength (RSS) and RSS indicator (RSSI) is vulnerable to low signal-to-noise ratios (SNRs) [24]. Second, since the LoRa signal is a narrowband signal, it cannot be very sharp in the time domain which makes accurately timing the arrivals of the LoRa signals at the gateway difficult. The timing resolution of the current LoRa devices is not sufficient for achieving accurate localization [24]. Some more efforts should be done to have the more accurate localization by adding other technologies. Anjum et al. [7] use regression and machine learning (ML) models for RSSI fingerprinting-based localization in LoRa networks. In the outdoor environments, the regression models can achieve around 77% accuracy and 46 m location error, and the machine learning models can achieve around 81.12% accuracy and 41.5 m location error—while, in the indoor environments, the regression models can achieve around 83.25% accuracy and 13.5 m location error, and the machine learning models can achieve around 87% accuracy and 11.78 m location error. The performance is expected to improve further by using deep learning models. Consequently, a deep neural network (DNN) is a useful technology to improve the system performance by pre-training a large set of labelled/unlabelled training data.

Some supervised learning-based localization techniques are presented [11,12,15,16,17,18,19]. Zhang et al. [11] initially proposed a four-layer DNN structure pre-trained by stacked denoising autoencoder (SDA) that is capable of learning reliable features from a large set of noisy samples from Wi-Fi signals. Xiao et al. [15] also proposed a BLE-based indoor localization pre-trained by a deep learning model, called a denoising autoencoder, to extract robust fingerprint patterns from received signal strength indicator measurements. To uniquely identify a LoRa device, Robyns et al. [16] designed a physical-layer fingerprinting, which can investigate and extract feature from radio signals, using supervised and zero-shot learning. Khatab et al. [17] proposed a fingerprinting method for indoor localization by using the autoencoder-based deep learning machine. Wang et al. [18] presented a deep residual sharing learning based system for WiFi based indoor localization with the channel state information (CSI). Decurninge et al. [19] proposed a CSI-based outdoor localization with a learning approach for a 5G-type MIMO system. Purohit et al. [12] use three different deep learning models (i.e., the Artificial Neural Network (ANN), Long Short-Term Memory (LSTM), and the Convolutional Neural Network (CNN)) for fingerprinting based location regression with LoRaWAN. An interpolation aided fingerprinting-based localization system architecture and a deep autoencoder method are proposed to effectively deal with a large number of missing samples/outliers.

The transfer learning technique is recently investigated in [8,9,10]. Pan et al. [8] provided a detailed survey of the transfer learning, to better understand the definitions and differences with inductive transfer learning, transductive transfer learning, and unsupervised transfer learning. Long et al. [9] proposed a domain invariant transfer kernel learning. The domain transfer learning involves two types of datasets. One is from the source domain by a number of labelled data, and the other one is from a target domain by a large amount of unlabelled data. Deng et al. [10] also proposed a new inductive transfer learning method.

It is noted that localization results using the semi-supervised transfer kernel learning are developed in [20,21,22,23]. Zou et al. [20] initially proposed an adaptive localization in dynamic indoor environment by using the transfer kernel learning. Qiu et al. [21] additionally presented an indoor localization approach by transfer learning from tracking outdoor motions to the indoor environment. Ghourchian et al. [22] presented a real-time indoor localization for Wi-Fi signals using the semi-supervised learning. The WiFi-only outdoor localization is proposed by Wang et al. [23], by holistically treating the large number of WiFi hotspot labels gather by crowdsensing. Wang et al. [23] utilized all of the labelled and unlabelled data for a given area using a semi-supervised manifold learning technique.

### 2.2. Motivation

As we know, there are only a few results investigating the outdoor localization by the semi-supervised learning technique. Most of the existing semi-supervised learning results are based on WiFi signals [22] or WiFi-hotspot labels gathered by crowdsensing [23].

The main problem is subdivided into two. the first problem is that the research about semi-supervised deep neural network positioning is mostly used in indoor environments. The second problem is that the accuracy of the outdoor positioning for LoRa is not high according to the related literature reviews of LoRa localization [2,3,4]. Combining the above issues, the research motivation is to improve the LoRa positioning accuracy in outdoor environments. We propose an outdoor grid segmentation localization scheme that can effectively reduce the LoRa outdoor localization error with a small number of labelled samples. Through the DNN model, the relationship between labelled samples can be learned, and more constraint virtual labelled samples based on constraint regression can be generated by the semi-supervised transfer learning. Through the iterative process, the virtual labelled samples are put into models so as to narrow the location area.

## 3. Preliminaries

This section describes the system model, the problem formulation, and the basic idea in Section 3.1–Section 3.3, respectively.

### 3.1. System Model

**Figure 1:**
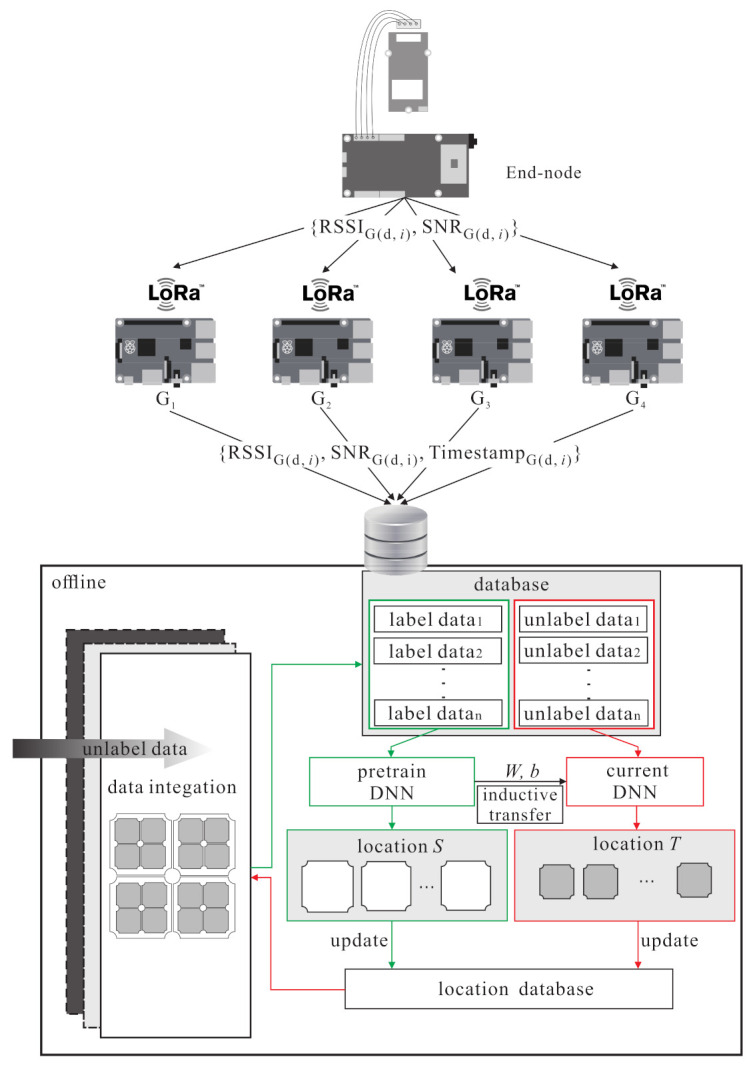
The LoRa localization system architecture using deep learning.

The system architecture of LoRaWAN-based localization with the semi-supervised learning is given in Figure 1. In the system, each LoRa end-node is equipped with the Semtech LoRa SX1276 module for the long range modem. Only a portion of the LoRa end-nodes are equipped with the Grove-GPS module armed with a SIM28 to acquire the GPS location information. In addition, a multi-gateway LoRaWAN is built on the Raspberry Pi 3, and each one is equipped with a Semtech LoRa SX1276 module.

When an end-node uses LoRaWAN protocol to send packets to gateways, the gateways can collect the sending time, RSSI, and SNR of the packet; at the same time, all gateways are synchronized by using the Greenwich mean time to get the packet arrival time. Then, the gateways forward the collected signal features via Wi-Fi and save data on Dropbox. Finally, the network server gets labelled samples and unlabelled samples from the Dropbox. Let *X* denote as a database, which includes *n* samples. Letting $x_{i}$ denote as the *i*-th sample, including *k* data from *g* gateways during a period of time *T* to collect RSSI, SNR, and timestamps (denoted as $\left(r_{i},snr_{i},t_{i}\right)$):

(1) $$X=\left\{x_{1},x_{2},\ldots ,x_{n}\right\}$$

(2) $$x_{i}=\left[\begin{array}{c} {\left[\left(r_{1},snr_{1},t_{1}\right),\left(r_{2},snr_{2},t_{2}\right),\ldots ,\left(r_{g},snr_{g},t_{g}\right)\right]}_{i_{1}}\\ {\left[\left(r_{1},snr_{1},t_{1}\right),\left(r_{2},snr_{2},t_{2}\right),\ldots ,\left(r_{g},snr_{g},t_{g}\right)\right]}_{i_{2}}\\ \ldots \\ \ldots \\ {\left[\left(r_{1},snr_{1},t_{1}\right),\left(r_{2},snr_{2},t_{2}\right),\ldots ,\left(r_{g},snr_{g},t_{g}\right)\right]}_{i_{k}} \end{array}\right]$$

When *n* samples are collected into database *X*, the main function is to divide the data collected by the Dropbox into two categories, labelled samples and unlabelled samples.

**Figure 2:**
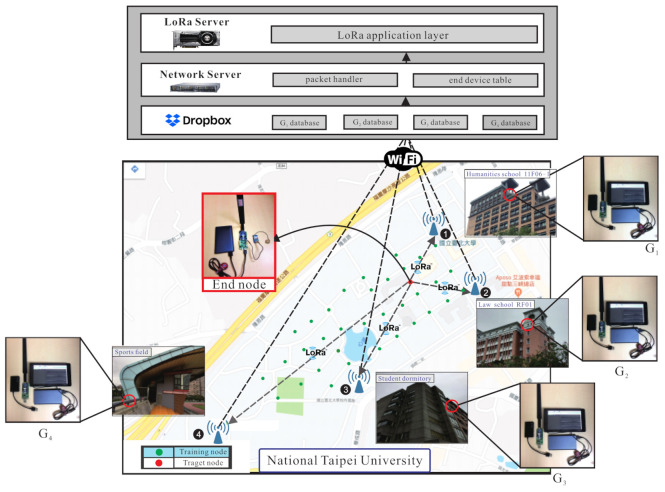
The outdoor environment of the large area.

In the training process, the environment is divided into large area and small area. The following description mainly focuses on the large area as shown in Figure 2. At the pre-training phase, each labelled samples from source domain corresponds to the true location $l_{m}^{s}$. The weight $W^{s}$ and bias $b^{s}$ of the source domain’s hidden layers (feature extractor) are frozen. The *m* hidden layers can be shown as follows:

(3) $$l_{m}^{s}=\left\{\left(x_{1}:y_{1}^{s}\right),\ldots ,\left(x_{i}:y_{i}^{s}\right),\ldots ,\;\left(x_{m}:y_{m}^{s}\right)\right\}$$

(4) $$y_{m}^{s}=\left\{\left(w_{1}^{s},w_{2}^{s},\ldots w_{m}^{s}\;|\;b_{1}^{s},b_{2}^{s},\ldots b_{m}^{s}\right)\in \Phi \right\}$$

The variable $y_{m}^{s}$ is defined as the label of instance $x_{m}^{s}$, and the variable Φ is defined as the source domain knowledge including the weight $W^{s}$ and bias $b^{s}$ of the frozen layers.

In the multi-kernel iteration phase, the unlabelled samples will use the labelled model knowledge Φ, and increase hidden layers to extract unlabelled features. In the first stage, labelled samples regression is used and each labelled location probability *p* is calculated to find the target domain. In the second stage, the virtual labelled samples are generated repeatedly until the error is less than the threshold so as to find the target classifier location $l^{t}$.

### 3.2. Problem Formulation

The proposed algorithm is performed on a forward neural networks architec- ture [11,15]. with the input sample *X*, the initial weight *W*, and the bias *b* in the hidden layers, and a nonlinear activation function σ.

The variable *f* represents as a feature vector, $f\in \left(RSSI_{i},SNR_{i},Timestamp_{i}\right)$. These signal feature values are extracted from an LoRa message sent from an end device to gateway $g_{i}$, where 1 ≤i≤j, *j* is the number of gateways.

We follow the same definition of transfer learning from [8]. Let $D_{s}=\left\{x_{i}^{s},y_{i}^{s},\theta \right\}$, $x_{i}^{s}$ be the source domain with the corresponding labelled data $y_{i}^{s}$, $y_{i}^{s}\in \left\{1,\ldots ,c\right\}$, and the variable θ is defined as the model parameter. Let $D_{t}=\left\{x_{i}^{t},\theta \right\}$, $x_{i}^{t}$ be the target domain with the unlabelled data under the same model parameter θ.

Based on training the labelled data in the source domain, the transfer learning can improve the accuracy of the unlabelled data in the target domain.

In this work, the source domain and target domain tasks are assumed to be the same, but $D_{s}\neq D_{t}$. Given the input $x_{i}^{s}$ and $x_{i}^{t}$, the training process goes through the hidden layers and nanonet layers to get the reconstruction output ${\hat{x_{i}}}^{s}$ and ${\hat{x_{i}}}^{t}$.

Minimizing the distribution divergence of the source domain and target domain is equal to minimizing the approximation error and optimizing the training accuracy of $D_{t}$, which is shown in the following:

(5) $$\begin{array}{c} \underset{W_{i}^{s},b_{i}^{s},W_{i}^{t},b_{i}^{t}}{\arg\min}\left\{\left(\sum_{i=1}^{n}{∥{\hat{x_{i}}}^{s}-{\hat{x_{i}}}^{t}∥}^{2}\right)-\left(\sum_{i=1}^{n}{∥x_{i}^{s}-x_{i}^{t}∥}^{2}\right)\right\}.\\ \;subject\;to\left\{\begin{array}{c} X=\left\{x_{1},x_{2},\ldots ,x_{n}\right\},n\geq 1\\ \;f\left(x:\theta \right)\neq 0\\ \;x^{s}\neq x^{t} \end{array}\right.\\ so\;as\;to\;maximize\;A \end{array}$$

The variables $W_{i}^{s},b_{i}^{s},W_{i}^{t}$, and $b_{i}^{t}$ are defined as the weight and bias values of the source domain and target domain respectively. *A* is defined as the accuracy of the unlabelled data in the labelled source domain. θ is defined as the model parameters.

When the training begins, the input sample $x_{i}$, through the initialization weight *W* and bias *b* in the hidden layers, uses the nonlinear activation function σ to lead to the nonlinear characteristics. The *m-th* hidden layer can be summarized as:

(6) $$hidden_{m}:\;\left(\hat{x}:f\right)=\sigma \left(W_{m}x+b_{m}\right)$$

### 3.3. Basic Idea

**Figure 3:**
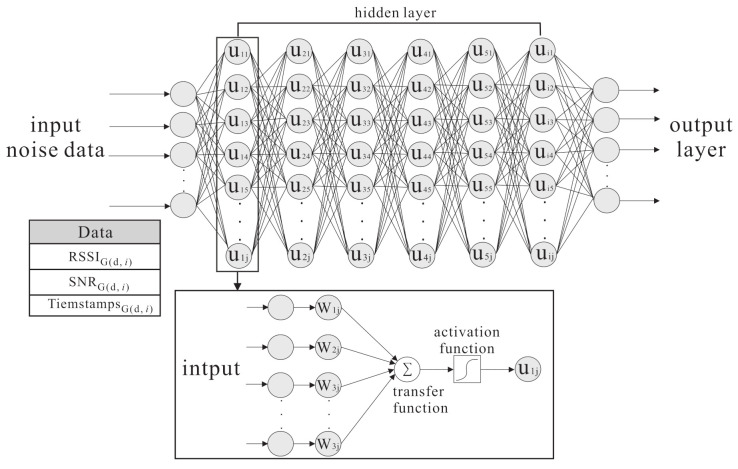
The Deep Neural Network system architecture.

**Figure 4:**
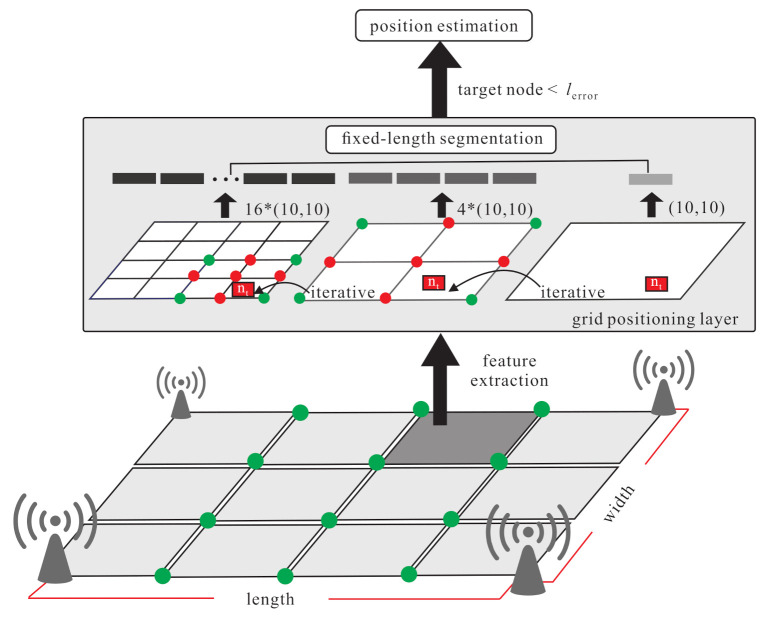
Basic idea of grid localization.

**Figure 5:**
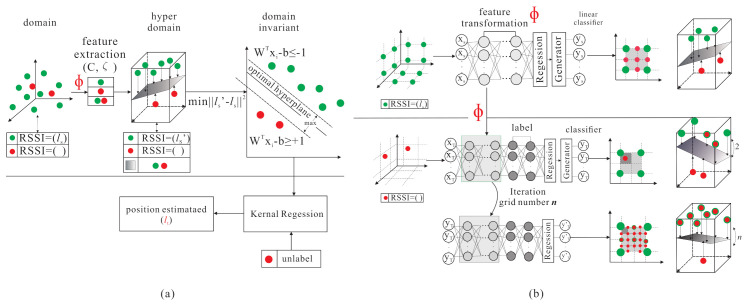
Comparison with (**a**) transfer kernel learning based on SVM; (**b**) transfer learning with grid segmentation based on DNN.

The basic idea contains two inspirations: improving the transfer learning location accuracy in the outdoor environment and reducing the large amount of data collection time. The proposed semi-supervised transfer learning uses the grid segmentation method to solve the problem as shown in Figure 3. The RSSI, SNR, and timestamps parameters are affected by a noisy environment. When the noisy parameters are put in the model, the weights and activation function are used in hidden layers in deep neural networks architecture to increase the effective feature parameters and reduce the noisy parameters effect. The purpose is to reduce the location error caused by the noisy parameters and increase the training accuracy. In Figure 4, the labelled samples are collected at the fixed distance of the grid. Therefore, when an end-node is within the area, the relationship between the end-node feature and the adjacent labelled samples can be used to find the area where the node is located. The grid segmentation concept is utilized so as to further generate virtual labelled samples and narrow the located area of the node. The virtual labelled points are generated repeatedly so as to narrow the range and minimize node positioning errors. Figure 5 demonstrates the differences between the SVM scheme and the proposed scheme.

## 4. The Proposed Outdoor Localization Scheme Using Semi-Supervised Transfer Learning with Grid Segmentation

This section presents the proposed outdoor localization scheme which uses semi-supervised transfer learning to predict the location of the unlabelled target for LoRaWANs. There are four phases in the proposed outdoor localization scheme, namely the source domain kernel pre-training phase, the kernel knowledge transferring phase, the source domain gird segmentation phase, and the grid segmentation fine-tuning phase, which are shown in the following subsections.

### 4.1. Source Domain Kernel Pre-Training Phase

**Figure 6:**
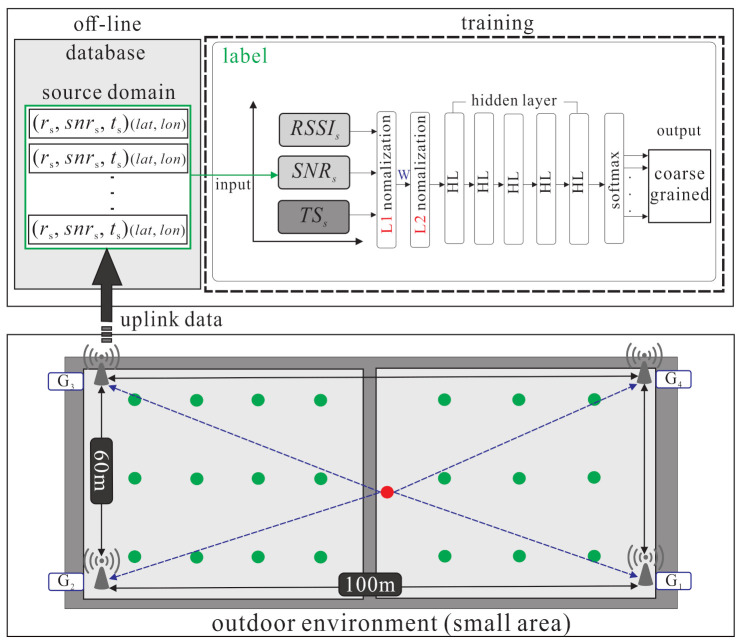
The source domain kernel pre-training phase.

The source domain kernel pre-training phase is shown in Figure 6. There is a pre-training for the source domain in DNN architecture. L1 $\left(\lambda_{1}\right)$ and L2 $\left(\lambda_{2}\right)$ are the normalization layers which can normalize parameters. Through supervised learning, the relationship between the parameters ($r_{i}^{s},snr_{i}^{s},t_{i}^{s}$) of the the source domain ($D_{s}$) and the classified results can be learned so as to get the feature of the source domain parameters and the regression kernel of each class. There are four steps in the source domain kernel pre-training phase.

**S1.**  The end-node transmits the sample $x_{i}$ to the gateways, then the gateways uplink the dataset to the database of the server. $x_{i}$ is shown in Equation (7):
  (7) $$x_{i}=\left[\begin{array}{c} {\left[\left(r_{1},snr_{1},t_{1}\right),\left(r_{2},snr_{2},t_{2}\right),\ldots ,\left(r_{g},snr_{g},t_{g}\right)\right]}_{i_{1}}\\ {\left[\left(r_{1},snr_{1},t_{1}\right),\left(r_{2},snr_{2},t_{2}\right),\ldots ,\left(r_{g},snr_{g},t_{g}\right)\right]}_{i_{2}}\\ \ldots \\ \ldots \\ {\left[\left(r_{1},snr_{1},t_{1}\right),\left(r_{2},snr_{2},t_{2}\right),\ldots ,\left(r_{g},snr_{g},t_{g}\right)\right]}_{i_{k}} \end{array}\right]$$
**S2.**  During the normalization layers (i.e., L1 $\left(\lambda_{1}\right)$ and L2 $\left(\lambda_{2}\right))$, L1 focuses on extracting the feature range of individual parameter and uses a minmaxscaler function to reduce the error between each parameters, where $x_{i}\to \tilde{x_{i}}$. The L1 normalization function is shown as follows:
  (8) $$\begin{array}{c} \lambda_{1}\;norm:\tilde{x}=\frac{x-x_{min}}{x_{max}-x_{min}} \end{array}$$
  Then, the labelled samples $\tilde{x_{i}}$ are fed into L2 $\left(\lambda_{2}\right)$. L2 focuses on extracting the feature range of all the parameters. The batch normalization function [25] has mini-batch data processing and considers the average means (μ) and standard deviation (σ) of all parameters so as to normalize the feature range, where $\tilde{x_{i}}\to \hat{x_{i}}$. The L2 normalization function is shown as follows:
  (9) $$\lambda_{2}\;norm:{\hat{x}}_{i}=\frac{{\tilde{x}}_{i}-\mu_{\tilde{x}}}{\sqrt{\sigma_{\tilde{x}}^{2}+\epsilon }}$$
  (10) $$\left(\mu_{\left(\tilde{x}\right)},\sigma_{\tilde{x}}^{2}\right)=\left(\frac{1}{m}\sum_{i=1}^{m}{\tilde{x}}_{i},\frac{1}{m}\sum_{i=1}^{m}{\left({\tilde{x}}_{i}-\mu_{\tilde{x}}\right)}^{2}\right)$$
**S3.**  This step puts the normalized $\hat{x_{i}}$ into the DNN architecture, and uses *m* hidden layers ($hidden_{m}$) and *l* encoder layers and *l* decoder layers to extract $D_{s}$ feature and each class regression kernel. The equations are shown as follows:
  (11) $${\tilde{x}}_{\left(s,t\right)}=\lambda_{2}\left(\lambda_{1}\left(Wx_{\left(s,t\right)}\right)\right)$$
  (12) $$hidden_{m}:\;\left({\hat{x}}_{\left(s,t\right)}:f\right)=\sigma \left(W{\tilde{x}}_{\left(s,t\right)}+b\right)$$
  In the pre-train model, the labelled samples $x_{i}$ and input data are got from the gateways, including $\left\{r_{i},snr_{i},t_{i}\right\}$.
**S4.**  After going through the supervised DNN model, the output class $\left(\hat{x_{i}},\hat{y_{i}^{s}}\right)$ is obtained, then the difference between the real class $\left(x_{i},y_{i}^{s}\right)$ and the output class $\left(\hat{x_{i}},\hat{y_{i}^{s}}\right)$ is calculated. The back propagation (BP) algorithm is used to update neurons in each hidden layer with both $W_{i}$ and $b_{i}$, to minimize the difference between $\left(x_{i},y_{i}^{s}\right)$ and $\left(\hat{x_{i}},\hat{y_{i}^{s}}\right)$. The equation is shown as follows:
  (13) $$\begin{array}{c} \underset{W_{i}^{s},b_{i}^{s}}{\arg\min}∥y_{i}^{s}-\hat{y_{i}^{s}}∥ \end{array}$$

**Figure 7:**
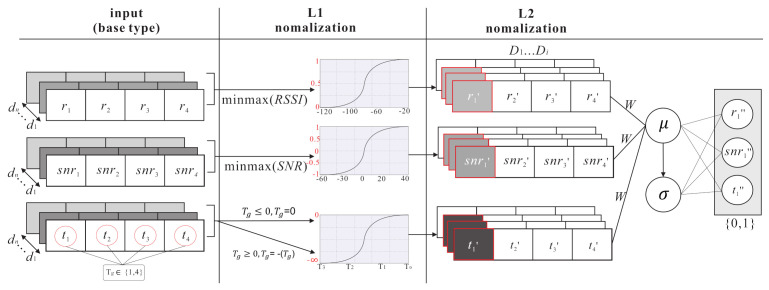
The normalization process for different parameters.

As shown in Figure 6, at **S1**, the end-node uses LoRaWANs to transmit *n* packets from one location $l_{1}$ to the gateways. When the gateways receive the packet, the gateways get timestamps from GMT and uplinks timestamps to the data server database. As shown in Figure 7, at **S2**, minmaxscaler function is used in L1. The batch normalization is used in L2, which considers multiple parameters {RSSI,SNR,TS}∈[0,1]. At **S3**, normalized parameters are put into the DNN model to get the output $\hat{y_{i}^{s}}$. At **S4**, the fine-tune process repeats by executing the BP algorithm to minimize the location error $\left(y_{i}^{s},\hat{y_{i}^{s}}\right)$.

### 4.2. Kernel Knowledge Transferring Phase

**Figure 8:**
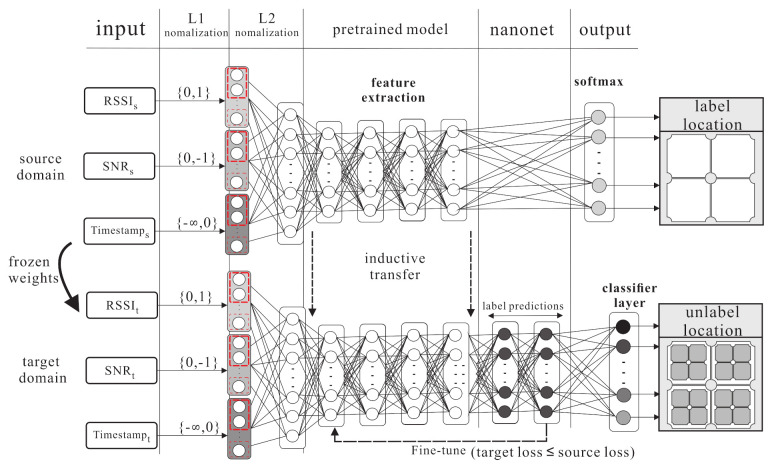
The kernel knowledge transferring phase.

The kernel knowledge transferring phase is shown in Figure 8. The knowledge is transferred from the source domain to the target domain. The source domain uses m+2l hidden layers, including *m* fully connected layers, *l* encoder layers, and *l* decoder layers to learn the largest area of labelled data. The softmax function is used to find the regression of labelled feature. The weight $W^{s}$ and bias $b^{s}$ of each layer are frozen. In the target domain, two hidden layers are added and all layers are fine-tuned to learn small area knowledge based on the source domain. There are two steps in the kernel knowledge transferring phase.

**S1.**  In this step, the softmax function uses the logistic regression for multi-class problems. The labelled class *y* is taken from the source domain, where $y\in \left\{1,\ldots ,c\right\}$. The probabilities of each class with instance ${\tilde{x}}^{s}$ can be estimated as follows:
  (14) $$l_{n}\left(\hat{x}\right)=\left[\begin{array}{c} p\left(y_{i}=1|\tilde{x};f\right)\\ p\left(y_{i}=2|\tilde{x};f\right)\\ .\\ .\\ .\\ p\left(y_{i}=c|\tilde{x};f\right) \end{array}\right]$$
**S2.**  The KL divergence (Kullback–Leibler divergence) is a non-symmetric measurement of the divergence between two probabilities of the embedded instance, which is between the source domain and target domain (denoted as $\left(\Phi_{s},\Phi_{t}\right)$). The probability is denoted as $\left(\left(P_{s},P_{t}\right)\right)$. The total statements can be written as $\left(\Phi_{s},\Phi_{t}\right)=d_{kl}\;(P_{s}\left|\right|P_{t})+d_{kl}\;(P_{t}\left|\right|P_{s})$, where $(P_{s}\left|\right|P_{t})\neq (P_{t}\left|\right|P_{s})$.

### 4.3. Source Domain Grid Segmentation phase

**Figure 9:**
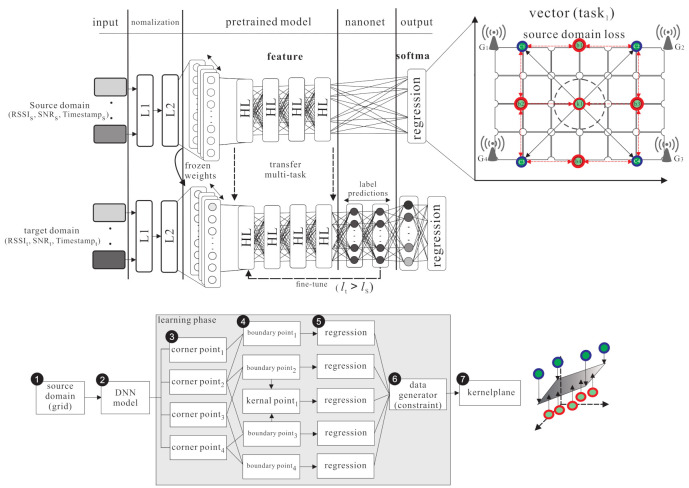
The source domain grid segmentation phase.

The source domain grid segmentation phase is shown in Figure 9. There are *n* labelled points (denoted as $s=\left\{s_{1},s_{2},\ldots ,s_{n}\right\}$) in the source domain. Select four points in the corner of the square area as corner points (denoted as $c=\left\{c_{1},c_{2},\ldots ,c_{4}\right\}$), then set the four points in the middle of any two corner points as the boundary points (denoted as $b=\left\{b_{1},b_{2},\ldots ,b_{4}\right\}$), finally select the point in the middle of the boundary points as the kernel point (demoted as $k_{1}$). The corner points are the input data $x^{s}$. The boundary points and the kernel point are the output class $y^{s}$. The DNN architecture is adopted as the training model. The data go through the hidden layers so as to extract the features and the softmax function is used to generate individual regression. There are three steps in the source domain grid segmentation phase.

**S1.**  Each labelled point has a corresponding feature (denoted as *f*). The labelled point, the corner point, the boundary point, the kernel point, and their corresponding features are denoted as $\left(s,f_{s}\right)$, $\left(c,f_{c}\right)$, $\left(b,f_{b}\right)$, and $\left(k,f_{k}\right)$, respectively. Those data are collected from a true noisy environment.
**S2.**  The DNN model is used to learn the boundary and kernel points from the corner points. This model uses supervised learning to generate boundary points from two constrained corners, $\left|f_{c_{1}},f_{c_{2}}\right|\to m_{1}$, and $\left|f_{m_{2}},f_{m_{3}}\right|\to k_{1}$, where $x_{\left\{c_{1},c_{2}\right\}}$ is the input data, $y_{m_{1}}$ is the output data, $W_{j}^{s}$ is the weight in the hidden layer *j*, $b_{j}^{s}$ is the bias of the hidden layer *j*, $v_{j}$ is the reconstruction input of the hidden layer *j*. $y_{m_{1}}$ can be derived from the followng equation:
  (15) $$y_{b_{1}}=\left(\sum_{i=1}^{n}W_{\left(1,n\right)}\right)x_{\left(c_{1},c_{2}\right)}+\sum_{i=1}^{n}b_{\left(1,n\right)}$$
**S3.**  The softmax function is used to calculate the regression probability of the output ($F_{s}^{\prime }=y_{m_{1}}$). KL divergence is used to calculate the loss function and sgd optimizer so as to fine-tune the weight $W^{s}$ and bias $b^{s}$ of each hidden layer $h_{i}$. Finally, to get the minimized error function, the frozen layer is added to transfer knowledge to the target domain..

### 4.4. Grid Segmentation Fine-Tuning Phase

**Figure 10:**
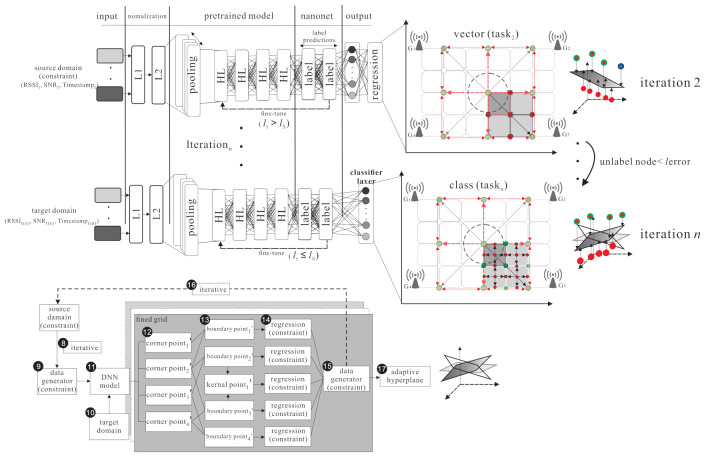
The grid segmentation fine-tuning phase.

The grid segmentation fine-tuning phase is shown in Figure 10. The grid segmentation process repeats so as to generate a large amount of constrained virtual labelled samples. After the grid segmentation and pre-training, the features $F_{s}$ can be obtained and then the knowledge, weight $w^{s}$, and bias $b^{s}$ to the target domain can be transferred. The unlabelled samples can learn the features $F_{s}$ so as to get the estimated location. Convert the surrounding boundary point and kernel point in the coarse grid to a new corner point $c_{s}^{\prime }$, then add two hidden layers to get more features from the unlabelled samples and get the new four boundary points (denoted as $b^{\prime }=\left\{b_{1}^{\prime },b_{2}^{\prime },\ldots ,b_{4}^{\prime }\right\}$) and new kernel point $k_{1}^{\prime }$ to calculate the new output features (denoted as $F_{s,t}$). The process repeats so as to fine-tune the grid size until the location errors of the unlabelled samples are less than the threshold.

**S1.**  Given unlabelled sample $X=\left\{x_{1},x_{2},\ldots ,x_{m}\right\}$, learn the weight $w_{n}^{s}$ and bias $b_{n}^{s}$ of each hidden layers $h_{n}$ from $F_{s}$ so as to fine-tune the coarse location as follows:
  (16) $$y_{m(1)}^{t}=\left(\sum_{i=1}^{n}W_{\left(1,n\right)}^{s}\right)x_{\left(1,m\right)}^{t}+\sum_{i=1}^{n}b_{\left(1,n\right)}^{s}$$
**S2.**  The grid is divided iteratively to get the new boundary points and new kernel points from the new corner points of the divided grid. The new corner points (denoted as $C^{\prime }=\left\{c_{1}^{{}^{\prime }},c_{2}^{{}^{\prime }},..,c_{4}^{{}^{\prime }}\right\}$) are the surrounding points of the original grid. Use the data generator to generate the corresponding data. Generate the new boundary points (denoted as $b^{\prime }=\left\{b_{1}^{{}^{\prime }},b_{2}^{{}^{\prime }},..,b_{4}^{{}^{\prime }}\right\}$) and the new kernel point (denoted as $k_{1}^{{}^{\prime }}$), and use the softmax function and KL divergence to derive the constraint regression. The weight $W_{n}^{s,t}$ and bias $b_{n}^{s,t}$ of the new hidden layer is frozen and $\left\{\left(W_{n}^{s,t},b_{n}^{s,t}\right)=F_{s,t}\right\}$. Finally, the fine-tuned location can be derived as follows:
  (17) $$y_{m_{1}}^{t}=\left(\sum_{i=1}^{n}W_{1,n}^{s,t}\right)x_{1,m}^{c^{\prime },b^{\prime },k^{\prime }}+\sum_{i=1}^{n}b_{1,n}^{s,t}$$

Assuming that there are m×n labels, after one iteration of fine tuning, there will be (2m−1)×(2n−1) labels and virtual labels. Hence, (2m−1)×(2n−1)−m×n virtual labels are added for training. After two iterations of fine-tuning, there will be (4m−3)×(4n−3) labels and virtual labels. Hence, (4m−3)×(4n−3)−m×n virtual labels are added for training. Similarly, after three iterations of fine-tuning, there will be (8m−7)×(8n−7) labels and virtual labels. Hence, (8m−7)×(8n−7)−m×n virtual labels are added for training. According to the above results, we can derive that, after k iterations of fine-tuning, there will be $\left(2km-2^{k}-1\right)\times \left(2kn-2^{k}-1\right)$ labels and virtual labels. $\left(2km-2^{k}-1\right)\times \left(2kn-2^{k}-1\right)-m\times n$ virtual labels are added for training. The extra computation and training cost for adding extra virtual labels is proportional to the number of extra virtual labels.

## 5. Experimental Results

**Figure 11:**
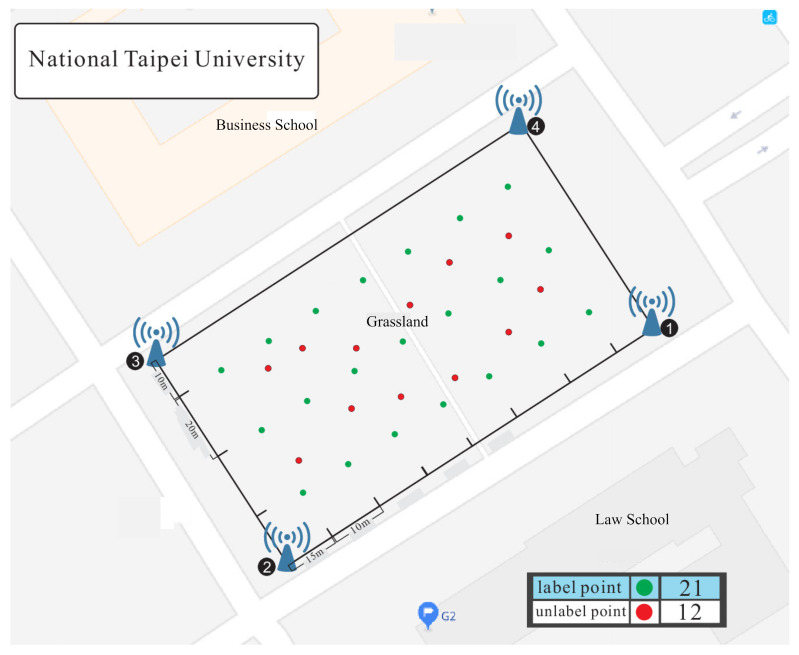
The experimental environment of the small area.

This section describes the environment configuration, setting of parameters, and experimental results. The experiments are performed in the campus of National Taipei University (NTPU). The campus is divided into small area (about 100×60 m ${}^{2}$) and large area (about 700×200 m ${}^{2}$) in the outdoor environment. Figure 2 shows the outdoor environment of the large area. The experiment uses one end-node and four gateways. Figure 11 shows the experimental environment of the small area. There are three scenarios in the experiments: the LL scenario (gateways and labelled points are all deployed in a larger area, i.e., lower gateway and labelled point density), the LS scenario (gateways are deployed in larger area and labelled points are deployed in smaller area, i.e., lower gateway density and higher labelled point density), and the SS scenario (gateways and labelled points are all deployed in smaller area, i.e., higher gateway and labelled point density). The end-node includes the Arduino DS1, Grove-GPS, 10,000 mAh mobile power bank, and LoRa modem Semtech sx1276. The gateways include Raspberry Pi3, LoRa modem sx1276 and use the RS232 interface. The LoRa spreading factor is set as SF9, the transmission power is set as 16 mW, the bandwidth is set as 125 KHz, the coding rate is set as 4/5, and the transmission time interval is set as 3 s. The GPU being used is Nvidia GTX 1050, the version of TensorFlow is 1.5.0, the version of Python is 3.6, and the operating system is Ubuntu 16.04 to implement a DNN architecture with 12 layers. There are 8116 data and 21 classes in the small area and there are 11,460 data and 34 classes in the large area. The learning rate is set as 0.0001, and the epoch is set as 5000. Three algorithms (the proposed scheme based on DNN, the original TKL scheme with SVM, and the adaptive TKL scheme with SVM) are adopted for experiment with single parameter *(RSSI)* denoted as S, and multiple parameters (RSSI, SNR, timestamps) denoted as M.

To evaluate the performance of the proposed localization scheme, the following performance metrics are observed in the experiments.

- Localization error: the mean difference between the real location and the predicted location.
- Data accuracy: the match rate of the output target data and the input data.
- Training time: the training time required to operate the entire system with different samples and different models.

### 5.1. Localization Error

**Figure 12:**
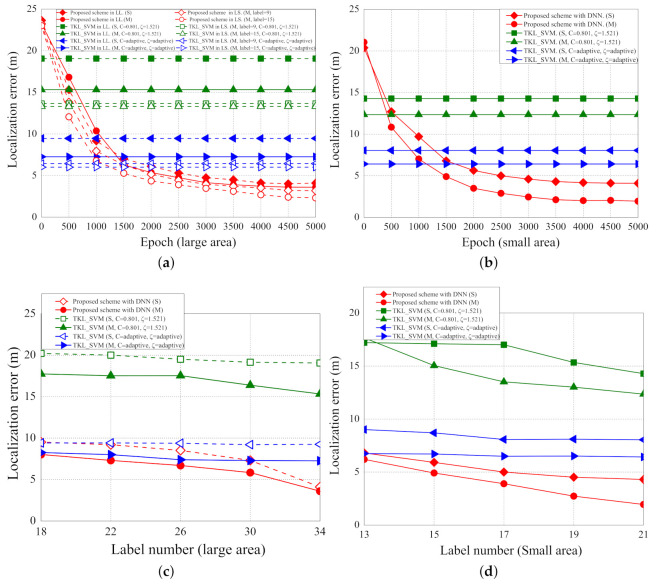
(**a**) Localization error (large area) vs. per epoch; (**b**) localization error (small area) vs. per epoch; (**c**) localization error (large area) vs. number of labelled samples; (**d**) localization error (small area) vs. number of labelled samples.

The localization errors with different epochs, scenarios, and number of labelled samples are shown in Figure 12a–d. Figure 12a shows the localization errors of LL and LS scenarios with different numbers of parameters and epochs. In LL with single parameter (LL(S)), the localization errors of the proposed schemes based on DNN, the original TKL scheme with SVM, and the adaptive TKL scheme with SVM are about 4.1, 19.06, and 9.47 m, respectively. In LL with multiple parameters (LL(M)), the localization errors of the proposed scheme based on DNN, the original TKL scheme with SVM, and the adaptive TKL scheme with SVM are about 3.6, 15.32, and 7.26 m, respectively. In LS with multiple parameters (LS(M)) and nine labelled points, the localization errors of the proposed scheme based on DNN, the original TKL scheme with SVM, and the adaptive TKL scheme with SVM are about 3.08, 13.65, and 6.44 m, respectively. In LS with multiple parameters and 15 labelled points, the localization errors of the proposed scheme based on DNN, the original TKL scheme with SVM, the adaptive TKL scheme with SVM are about 2.32, 13.65, and 6.01 m, respectively.

Figure 12b shows the localization errors of SS scenario with a different number of parameters and epochs. In SS with single parameter (SS(S)), the localization errors of the proposed schemes based on DNN, the original TKL scheme with SVM, and the adaptive TKL scheme with SVM are about 4.1, 14.28, and 8.05 m, respectively. In SS with multiple parameters (SS(M)), the localization errors of the proposed scheme based on DNN, the original TKL scheme with SVM, and the adaptive TKL scheme with SVM are about 1.94, 12.35, and 6.42 m, respectively.

As the epoch increases, the localization errors of the proposed scheme decreases because more epochs of training can achieve higher accuracy of training results and hence the localization errors decrease. The iterations of epoch do not affect the localization errors of the two SVM based schemes because the two SVM based schemes are not based on the deep learning architecture. The localization errors in the SS scenario with multiple parameters are the least because, in such a scenario, the labelled points are closer to each other and more parameters can achieve more accurate training results and thus the localization errors decrease. The proposed scheme performs better than the two SVM based schemes because we use the grid segmentation to fine-tune the predicting locations.

Figure 12c shows the localization errors of LL scenarios with a different number of labelled samples (from 18 to 34 samples). In the LL scenario with single parameter (S), the reduced localization errors (localization errors of 18 samples minus localization errors of 34 samples) of the proposed schemes based on DNN, the original TKL scheme with SVM, and the adaptive TKL scheme with SVM are about 5.32, 1.16, and 0.17 m, respectively. In the LL scenario with multiple parameters (M), the reduced localization errors of the proposed scheme based on DNN, the original TKL scheme with SVM, and the adaptive TKL scheme with SVM are about 4.42, 2.42, and 1 m, respectively. As the number of labelled samples increases, the reduced localization errors increase (i.e., localization errors decrease) because the labelled points are closer to each other and thus the localization errors decrease.

Figure 12d. shows the localization errors of SS scenario with a different number of labelled samples (from 13 to 21 samples). In the SS scenario with single parameter (S), the reduced localization errors of the proposed schemes based on DNN, the original TKL scheme with SVM, and the adaptive TKL scheme with SVM are about 2.5, 2.93, and 0.96 m, respectively. In the SS scenario with multiple parameters (M), the reduced localization errors of the proposed scheme based on DNN, the original TKL scheme with SVM, and the adaptive TKL scheme with SVM are about 4.2, 5.31, and 0.32 m, respectively.

**Figure 13:**
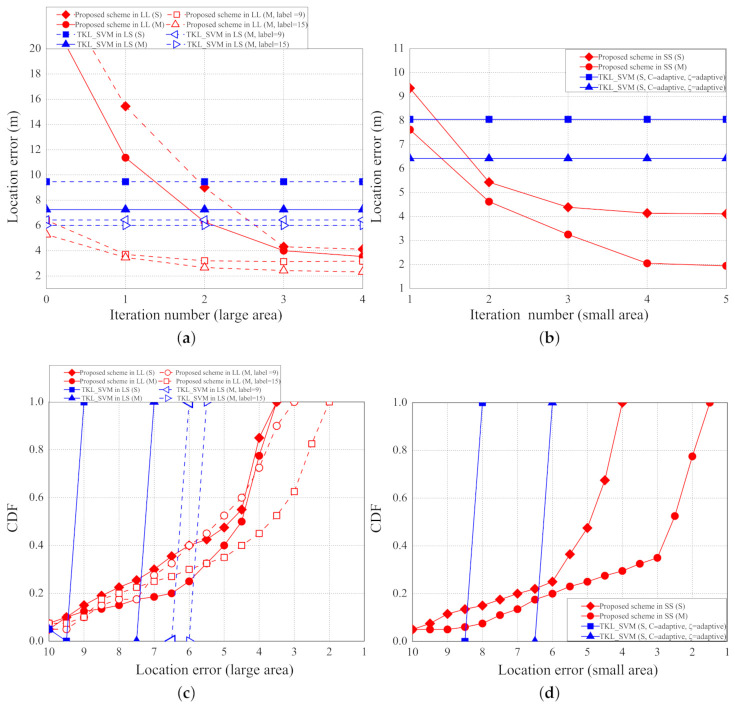
(**a**) Localization error (LL,LS) vs. number of iterations; (**b**) localization error (SS) vs. number of iterations; (**c**) CDF vs. localization error (LL,LS); (**d**) CDF vs. localization error (SS).

The localization errors with a different number of fine-tuned iterations are shown in Figure 13a,b. As the number of iterations increases, the localization errors of the proposed scheme decrease because we use grid segmentation to fine-tune the predicting locations. The number of iterations do not affect the localization errors of the two SVM based schemes because the two SVM based schemes do not perform grid segmentation to fine-tune the predicting locations. When the number of iterations is greater than 4, the localization errors converge because the grid size is too small to tell the differences between different virtual labelled points.

The CDF of localization errors in the scenarios of large area (LL and LS) and small area (SS) are shown in Figure 13c,d, respectively. In the proposed scheme, more than 50% of the localization errors are less than 5 m; in the two SVM-based scheme, 100% of the localization errors are greater than 6 m. In SS scenario with multiple parameters, 80% of the localization errors of the proposed scheme are less than 6 m.

### 5.2. Location Accuracy

**Figure 14:**
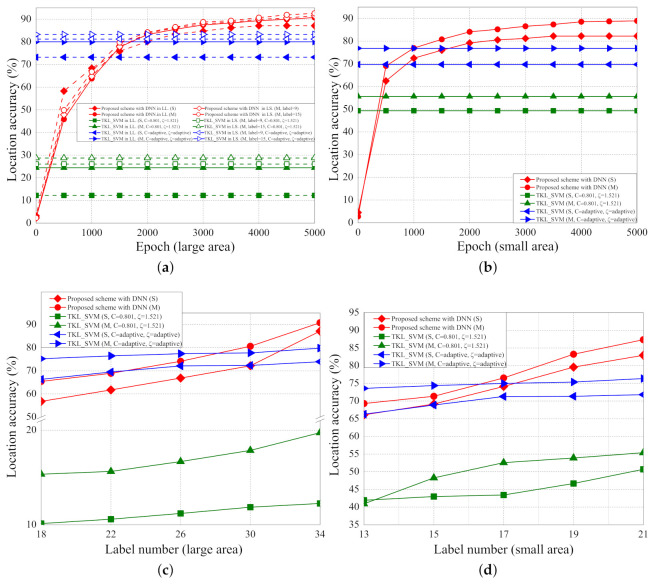
(**a**) Location accuracy (LL,LS) vs. per epoch; (**b**) location accuracy (SS) vs. per epoch; (**c**) location accuracy (LL,LS) vs. number of labelled samples; (**d**) location accuracy (SS) vs. number of labelled samples.

The location accuracies with different epochs, scenarios, and number of labelled samples are shown in Figure 14a–d. Figure 14a shows the location accuracies of LL and LS scenarios with different numbers of parameters and epochs. In LL with a single parameter, the localization accuracies of the proposed schemes based on DNN in 5000 iterations of epoch, the original TKL scheme with SVM, and the adaptive TKL scheme with SVM are about 87.14%, 12.22%, and 73.23%, respectively. In LL with multiple parameters, the localization accuracies of the proposed schemes based on DNN in 5000 iterations of epoch, the original TKL scheme with SVM, and the adaptive TKL scheme with SVM are about 90.78%, 25.44%, and 80.74%, respectively. In LS with multiple parameters and nine labelled points, the localization accuracies of the proposed schemes based on DNN in 5000 iterations of epoch, the original TKL scheme with SVM, and the adaptive TKL scheme with SVM are about 91.08%, 26.07%, and 81.24%, respectively. In LS with multiple parameters and 15 labelled points, the localization accuracies of the proposed schemes based on DNN in 5000 iterations of epoch, the original TKL scheme with SVM, and the adaptive TKL scheme with SVM are about 91.61%, 28.74%, and 83.31%, respectively.

Figure 14b shows the location accuracies of SS scenarios with different number of parameters and epochs. In an SS scenario with single parameter (SS(S)), the localization accuracies of the proposed schemes based on DNN, the original TKL scheme with SVM, and the adaptive TKL scheme with SVM are about 82.23%, 49.25%, and 69.85% m, respectively. In an SS scenario with multiple parameters (SS(M)), the localization accuracies of the proposed scheme based on DNN, the original TKL scheme with SVM, and the adaptive TKL scheme with SVM are about 88.94%, 55.65%, and 76.85% m, respectively.

As the epoch increases, the localization accuracies of the proposed scheme also increase because more epochs of training can achieve higher accuracy of training results and hence the localization accuracy increases. The iterations of epoch do not affect the localization accuracies of the two SVM based schemes because the two SVM based schemes are not based on the deep learning architecture. The localization accuracies in the environment of LS with multiple parameters and 15 labelled points are the best because, in such environment, the labelled points are closer to each other and more parameters can achieve more accurate training results and thus the localization accuracies are the highest

Figure 14c shows the location accuracies of LL scenarios with different numbers of labelled samples (from 18 to 34 samples). In LL scenario with single parameter (S), the increased localization accuracies (localization accuracies of 34 samples minus localization accuracies of 18 samples) of the proposed schemes based on DNN, the original TKL scheme with SVM, and the adaptive TKL scheme with SVM are about 30.35%, 2.12%, and 7.62%, respectively. In an LL scenario with multiple parameters (M), the increased localization accuracies of the proposed scheme based on DNN, the original TKL scheme with SVM, and the adaptive TKL scheme with SVM are about 25.36%, 4.39%, and 4.62%, respectively. As the number of labelled samples increases, the increased location accuracies also increase (i.e., location accuracies increase) because the labelled points are closer to each other and thus the localization accuracies increase.

Figure 14d. shows the location accuracies of SS scenario with different numbers of labelled samples (from 13 to 21 samples). In the SS scenario with single parameter (S), the increased localization accuracies of the proposed schemes based on DNN, the original TKL scheme with SVM, and the adaptive TKL scheme with SVM are about 16.83%, 8.76%, and 5.53%, respectively. In an SS scenario with multiple parameters (M), the increased localization accuracies of the proposed scheme based on DNN, the original TKL scheme with SVM, and the adaptive TKL scheme with SVM are about 18.05%, 14.44%, and 2.78%, respectively.

**Figure 15:**
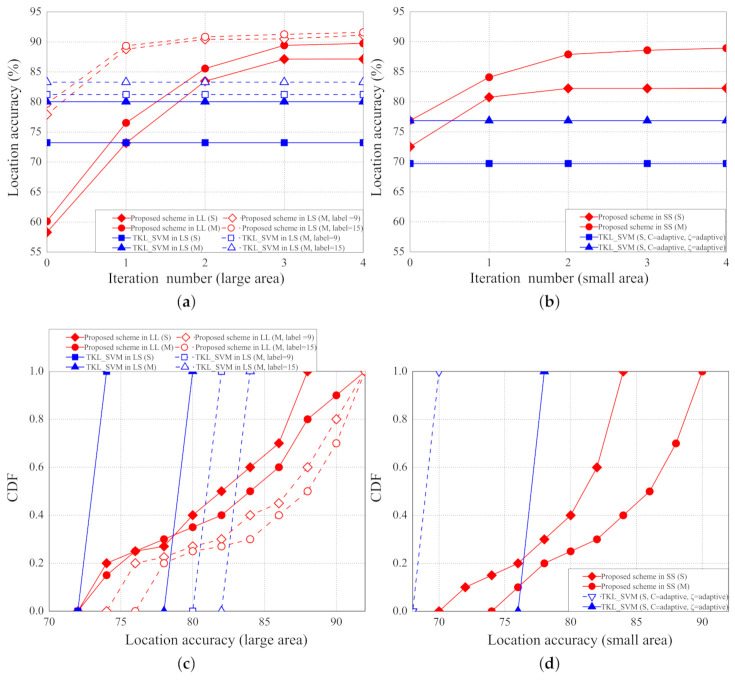
(**a**) Location accuracy vs. number of iterations (LL,LS); (**b**) location accuracy vs. number of iterations (SS); (**c**) CDF vs. location accuracy (LL,LS); (**d**) CDF vs. location accuracy (SS).

The location accuracies with different number of iterations are shown in Figure 15a,b. As the number of iterations increases, the localization accuracies of the proposed scheme increase because we use grid segmentation to fine-tune the training results. The number of iterations do not affect the localization accuracies of the two SVM based schemes because the two SVM based schemes do not perform grid segmentation to fine-tune the training results. When the number of iterations is greater than 3, the location accuracies converge because the grid size is too small to tell the differences between different virtual labelled points.

The CDF of localization accuracies in the scenarios of large area (LL and LS) and small area (SS) are shown in Figure 15c,d, respectively. In the proposed scheme, more than 60% of the location accuracies are higher than 80%; in the two SVM-based scheme, 100% of the location accuracies are lower than 84%. In an LL scenario with multiple parameters, more than 75% of the localization errors of the proposed scheme are higher than 80%.

### 5.3. Training Time

**Figure 16:**
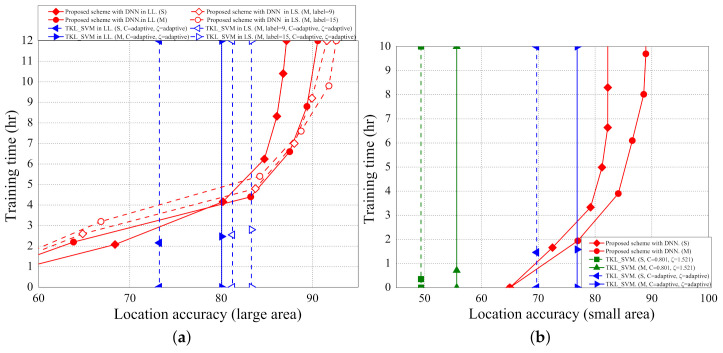
(**a**) The training time (LL,LS) vs. location accuracy; (**b**) the training time (SS) vs. location accuracy.

Figure 16a,b shows the impact of training time on location accuracies in the scenarios of large area and small area, respectively. As the training time increases, the location accuracies of the propose scheme also increase. The training time does not affect the localization accuracies of the two SVM based schemes because the two SVM based schemes are not based on the deep learning architecture. The localization accuracy in the scenario of small area converges faster than that in the scenario of large area because the sampled data in the small area is less variant.

**Figure 17:**
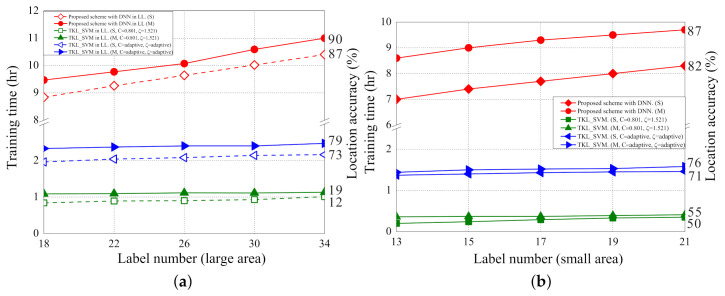
(**a**) The training time (LL,LS) vs. number of labelled samples; (**b**) the training time (SS) vs. number of labelled samples.

Figure 17a,b shows the impact of the number of labelled samples to training time in the scenarios of large area and small area, respectively. As the number of labelled samples increases, the training time also increases because more input data cause more training time in the deep learning architecture. The number of labelled samples has less effect on the training time of the two SVM based schemes because the two SVM based schemes are based on machine learning. Overall, the proposed localization scheme takes more time in training but can achieve higher data accuracies as opposed to the two SVM-based schemes.

## 6. Conclusions

A novel grid segmentation localization scheme using a semi-supervised transfer learning for LoRaWANs is proposed in this paper. The proposed scheme uses three signal features, RSSI, SNR, and timestamps, for training and learns grid segmentation knowledge from the source domain and transfer the knowledge to the target domain. A number of virtual labelled samples are generated by figuring out the relationship of labelled and unlabelled samples. With the labelled-unlabelled samples relationship, the target model is repeatedly fine-tuned by adding more new virtual labelled samples. The proposed scheme is implemented on the campus of National Taipei University. Experiment results show that the proposed scheme can decrease the localization errors and improve the location accuracies in an outdoor environment for LoRaWANs. The proposed localization scheme using semi-supervised transfer learning with grid segmentation can also be implemented in other wireless networks based on other deep learning architectures (e.g., WiFi networks on the autoencoder architecture).

## Data Availability

Data sharing is not applicable to this article.
